# Stability of *Staphylococcus aureus* Phage ISP after Freeze-Drying (Lyophilization)

**DOI:** 10.1371/journal.pone.0068797

**Published:** 2013-07-02

**Authors:** Maia Merabishvili, Chris Vervaet, Jean-Paul Pirnay, Daniel De Vos, Gilbert Verbeken, Jan Mast, Nino Chanishvili, Mario Vaneechoutte

**Affiliations:** 1 Laboratory for Molecular and Cellular Technology, Queen Astrid Military Hospital, Brussels, Belgium; 2 Laboratory Bacteriology Research, Department Clinical Chemistry, Microbiology and Immunology, Faculty of Medicine and Health Sciences, University of Ghent, Ghent, Belgium; 3 R&D Department, Eliava Institute of Bacteriophage, Microbiology and Virology, Tbilisi, Georgia; 4 Laboratory of Pharmaceutical Technology, Faculty of Pharmaceutical Sciences, University of Ghent, Ghent, Belgium; 5 Electron Microscopy Unit, Veterinary and Agrochemical Research Centre, Brussels, Belgium; Naval Research Laboratory, United States of America

## Abstract

*Staphylococcus aureus* phage ISP was lyophilized, using an Amsco-Finn Aqua GT4 freeze dryer, in the presence of six different stabilizers at different concentrations. Stability of the lyophilized phage at 4°C was monitored up to 37 months and compared to stability in Luria Bertani broth and physiological saline at 4°C. Sucrose and trehalose were shown to be the best stabilizing additives, causing a decrease of only 1 log immediately after the lyophilization procedure and showing high stability during a 27 month storage period.

## Introduction

Phage therapy has attracted intense renewed scientific and public interest, mostly as a consequence of the increasing problem of antibiotic-resistant strains emerging worldwide. While the number of phage research articles and well-defined trials is increasing [1], [2], there is still a lack of information about proper pharmaceutical formulations and preservation conditions guaranteeing effective outcome of phage therapy.

In general, phage preparations applied with therapeutic aims are produced in liquid formulation, and the storage period of such preparations, at 4°C, is considered as limited to one year. Recently, different novel approaches have been described, such as encapsulating phages into different biodegradable materials [5], [6], [7] and aerosols [8], [9]. These approaches mostly involve the use of lyophilized bacteriophages. The methods of lyophilization described in these studies vary significantly with regard to both stabilizers and freeze-drying regimes. The most detailed studies on lyophilization of bacteriophages date back to the 70′s of the last century [10], [11], [12], [13], [14] (Table 1). In this study, we optimized the lyophilization process of the therapeutically important bacteriophage ISP, active against strains of *Staphylococcus aureus* including MRSA, and explored in more detail the properties of different modern pharmaceutically acceptable stabilizers.

**Table 1:** Overview of bacteriophage lyophilization studies.

Author, year	Bacterial species, phage (number of)	Lyophilisation method	Additives	Storage t° (°C)	Duration	Initial titer (log)	Remaining titer (log)	% loss
Clark, 1962 [2123]	Different phages (22)	Weiss, 1957 [27]	50% skim milk	4, 26	2 years	7–10	6–9	NS
Davies and Kelly, 1969 [12]	*Corynebacterium* phage H1	Greaves and Davies, 1965 [28]	20% peptone	RT	3 months	NS	NS	25
Davies and Kelly, 1969 [12]	*Corynebacterium* phage H1	Greaves and Davies, 1965 [28]	20% peptone +10% sucrose	RT	3 months	NS	NS	18
Davies and Kelly, 1969 [12]	*Corynebacterium* phage H1	Greaves and Davies, 1965 [28]	20% peptone +10% sucrose+2% sodium glutamate	RT	3 months	NS	NS	46
Steel *et al*., 1969 [14]	*Escherichia coli* phage T4	Steel et al., 1969 [14]	Peptone	NA	NA	6–8	NS	18–99
Carne and Greaves, 1974 [10]	*Corynebacterium* phages (14)	Greaves and Davies, 1965 [28]	20% peptone +10% sucrose+2% sodium glutamate	min 25	2.5 years	7–10	7–10	NS
Cox et al., 1974 [11]	*E. coli* phage T3	Cox and Heckly, 1973 [29]	Luria-Bertani broth	NS	NS	12	NS	87
Cox et al., 1974 [11]	*E. coli* phage T3	Cox and Heckly, 1973 [29]	0.05 M sucrose	NS	NS	12	NS	51
Cox et al., 1974 [11]	*E. coli* phage T7	Cox and Heckly, 1973 [29]	Luria-Bertani broth	NS	NS	12	NS	99
Cox et al., 1974 [11]	*E. coli* phage T7	Cox and Heckly, 1973 [29]	0.05 M sucrose	NS	NS	12	NS	87
Shapira and Kohn, 1974 [13]	*E. coli* phage T4	Lion and Bergman, 1961 [30]	None	min 20	0–7 days	9.9	NS	99.8500
Shapira and Kohn, 1974 [13]	*E. coli* phage T4	Lion and Bergman, 1961 [30]	Glucose 4000 µg/ml	min 20	0–7 days	9.9	NS	99.9500
Shapira and Kohn, 1974 [13]	*E. coli* phage T4	Lion and Bergman, 1961 [30]	Gelatine 20 µg/ml	min 20	0–7 days	9.9	NS	99.9600
Shapira and Kohn, 1974 [13]	*E. coli* phage T4	Lion and Bergman, 1961 [30]	Gelatine 200 µg/ml	min 20	0–7 days	9.9	NS	99.9200
Shapira and Kohn, 1974 [13]	*E. coli* phage T4	Lion and Bergman, 1961 [30]	PBS	min 20	0–7 days	9.9	NS	99.9920
Shapira and Kohn, 1974 [13]	*E. coli* phage T4	Lion and Bergman, 1961 [30]	NaCl 0.9%	min 20	0–7 days	9.9	NS	99.9997
Zierdt, 1988 [24]	*Staphylococcus aureus*typing phages (25)	Zierdt, 1959 [31]	100% skim milk	min 20	12–18 years	2–3	1–3	NS
Ackermann *et al*., 2004 [22]	Caudovirales (12)	NS	50% glycerol	4	20 years	NS	viable	NS
Puapermpoonsiri *et al*., 2009 [6]	*Staphylococcus aureus* Siphovirus	Puapermpoonsiri *et al.,*2009 [6]	1 M Tris–HCl, 0.1 M NaCl,8 mM MgSO_4_, 0.1 g/L gelatin	NA	NA	9.7, 7.8, 3.7	++p, +p, −p a	NS
Puapermpoonsiri *et al*., 2010 [7]	*S. aureus* Siphovirus and*P.aeruginosa* Myovirus	Puapermpoonsiri *et al.,*2010 [7]	0.1 M and 0.5 M sucrose,1% and 5% PEG 6000	4	2, 7, 14, 30 days	8	From ++p to +p^a^	NS
Alfadhel *et al*., 2011 [26]	*S. aureus* phage	Puapermpoonsiri *et al.,*2010 [7]	1 ml HPMC +/−1% w/v mannitol	4	6–12 months	8 per insert	5–6	NS
Anany *et al.*, 2011 [25]	*E. coli* phage T4	Anany *et al.,* 2011 [25]	0.5% maltose	NA	NA	NS	NS	NS^b^
Anany *et al*., 2011 [25]	*E. coli* phage T4	Anany *et al.,* 2011 [25]	5% maltose	NA	NA	NS	NS	NS^b^
Anany *et al*., 2011 [25]	*E. coli* phage T4	Anany *et al.,* 2011 [25]	0.3% soluble starch	NA	NA	NS	NS	NS^b^
Anany *et al*., 2011 [25]	*E. coli* phage T4	Anany *et al.,* 2011 [25]	None	NA	NA	NS	NS	NS^b^

NA-non applicable, NS: not specified, RT: room temperature.

a : ++p: Confluent lysis (fragmented bacterial lawn), +p: Individual plaques too many to count (>400 per plate), −p:No plaques.

b : no significant reduction P<0.05.

## Materials and Methods

### Bacteria and Bacteriophages

Phage ISP is maintained in the phage collection of the LBR (University of Ghent, Belgium) since 2002 and was received from the Eliava IBMV (Tbilisi, Georgia). For the propagation of the ISP phage, we used *Staphylococcus aureus* strain ‘13 S44 S9’, isolated from a burn wound at the Brussels Burn Wound Centre (Queen Astrid Military Hospital, Brussels, Belgium) in 2006.

### Phage Propagation and Enumeration

The bacterial strain and the phage were cultured in Select Alternative Protein Source Luria Bertani (APS LB) (Becton Dickinson, Erembodegem, Belgium) media. The agar overlay method with modifications as described earlier [15] was used to obtain high titer (11 log pfu/ml) phage lysates. Briefly, 1 ml of phage suspension containing 4 log pfu of ISP was mixed with 3.0 ml of molten (45°C) APS LB top agar (0.7%) and 0.1 ml of a host bacterial suspension (end concentration of 8 log cfu/ml). This mixture was plated onto Petri dishes, filled with a bottom layer of 1.5% APS LB agar and incubated at 37°C for 16–18 h. The top agar layer was scraped off and centrifuged for 20 min at 6 000 *g*. The supernatant was filtered through a 0.45 µm membrane filter (Sartorius Stedim Biotech, Göttingen, Germany).

The obtained phage lysate was ultracentrifuged at 25000 *g* for 1 h at 4°C and the pellet was resuspended in the same volume of a 0.9% NaCl solution. Phage particles were enumerated by the agar overlay method [15]. Briefly, decinormal serial dilutions (from log(0) to log(−10)) of the bacteriophage suspension were prepared. One ml of each dilution was mixed with 3.0 ml of molten (45°C) 0.7% LB top agar and 0.1 ml of a host bacterial suspension (end concentration of 8 log cfu/ml) and plated in triplicate onto 90 mm diameter Petri dishes (Plastiques Gosselin, Menen, Belgium), filled with a bottom layer of 1.5% LB agar and incubated for 18–24 h at 37°C. To estimate the original bacteriophage concentration, plates with 100–1000 plaques were counted. Each titration was performed three times. The mean was then calculated for the triplicate plates and for each titration.

### Lyophilization

Bacteriophage solutions were prepared using the following six stabilizers with two different concentrations: 0.1 M and 0.5 M for sucrose, trehalose, mannitol and glycine (BASF, Ludwigshafen, Germany) and 1% and 5% for PVP (polyvinylpyrrolidone) (BASF, Ludwigshafen, Germany) and PEG 6000 (polyethylene glycol) (Fagron, Waregem, Belgium).

Phage stock solution (11 log pfu/ml) was diluted in stabilizers to a final titer of 8 log pfu/ml for the first series of tests and 9 log pfu/ml for the second series. Ten-ml freeze-drying vials, containing one ml of phage solutions, were lyophilized in an Amsco-Finn Aqua GT4 freeze dryer (Amsco, Hürth, Germany), using the following lyophilization cycle: prior to loading the vials into the freeze dryer the shelves were pre-cooled to −5°C. After loading, the vials were cooled to −30°C at a cooling rate of 1°C/min. A temperature of −30°C was maintained during 80 min to ensure complete solidification of the material. Primary drying was performed at −30°C and 300 µbar during 1000 min. For secondary drying the temperature was gradually increased from −30 to 25°C over a period of 550 min, followed by an isothermal period at 25°C during 360 min. The pressure during secondary drying was maintained at 300 µbar. After freeze-drying, the vials were sealed at atmospheric pressure using Omniflex stoppers (Helvoet Pharma, Alken, Belgium). Twenty replicates were made for each concentration of each stabilizer.

Lyophilized phages were stored at 4°C and checked for stability after different periods.

### Phage Stability Tests

Lyophilized phages, phage particles suspended in LB broth (10 log pfu/ml) and in non-buffered 0.9% NaCl solution (9 log pfu/ml), stored at 4°C were monitored for the maintenance of stability during a maximum of a 37 month period. In case of lyophilized phages, the freeze-dried cakes were reconstituted by adding 1 ml of sterile 0.9% NaCl solution and serial dilutions were performed. Phage enumeration tests were performed in triplicate by agar overlay method [15], as described above.

### Comparison of Phage Genomes

DNA homology of bacteriophages ISP (GenBank: FR852584.1) and Sb-1 (GenBank: HQ163896.1) was compared by EMBOSS stretcher [16].

### Transmission Electron Microscopy

Resuspended lyophilized samples of ISP were analyzed by transmission electron microscopy as described in Merabishvili *et al.* [15]. The samples were analyzed using a Technai Spirit transmission electron microscope (FEI, Eindhoven, The Netherlands) operating at 120 kV. Micrographs were recorded using a bottom-mounted digital camera (Eagle, 4×4K, FEI).

## Results and Discussion

ISP is a virulent myovirus, representative of morphotype A1 [15] widely used since decades for therapeutic purposes in Georgia. ISP represents the main component of the Intravenous Staphylococcal Phage preparation produced in the 70–80’s by the Eliava IMBV. Lately, ISP was included in a quality-controlled prepared phage cocktail BFC-1, used for a pilot safety study in Belgium [15], and its genome sequence has been determined [17]. According to different studies, ISP is active against 86% [17] – 91% [15] of the clinical isolates of *S. aureus*, including MRSA. ISP as a broad-host-range phage is considered as an appropriate candidate for production of monoclonal phage preparations.

Genome sequence analysis revealed that *Myovirus* ISP is closely related to the ‘Twort-like viruses’ [17]. The genome of ISP is 99.5% and 90.6% identical to the genome of phages G1 [18] and Sb-1 [19], respectively, as determined by EMBOSS stretcher [16], [17]. The genome sequence analysis of ISP confirmed the lytic character of ISP and the absence of toxin genes [17].

Because, based on these different characteristics, ISP is a phage with high therapeutic potential, it seemed to be an appropriate candidate to study the lyophilization optimization process, applicable for long storage of bacteriophages and for their further incorporation in different pharmaceutical formulations.

In general lyophilization process generates a variety of freezing and drying stresses, such as solute concentration, formation of ice crystals, pH changes, etc. All of these stresses can cause destabilization of the processed biological material or biomolecules to various degrees [20].

**Figure 1:**
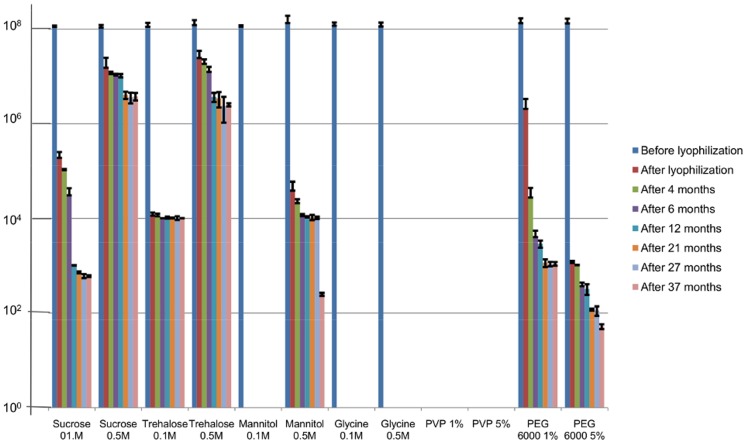
Stability of ISP (8 log pfu/ml) in six different stabilizers after freeze-drying procedure and storage at 4°C. The results are the mean values of three titrations. Standard deviations are indicated.

Therefore special stabilizers must be added to protect these fragile systems from freezing stress (cryoprotectant) or drying stress (lyoprotectant) and also to increase its stability upon storage [21]. Six stabilizers representing various groups of common stabilizers were chosen for the first set of tests performed on ISP phage. Each stabilizer was applied in two different concentrations. Figure 1 presents the stability of the ISP lyophilisates, for each stabilizer. PVP, representative of polymer excipients, inactivated the phage completely at both concentrations (1 and 5%), even prior to lyophilization. Stabilization of proteins by polymers is generally attributed to preferential exclusion, surface activity, steric hindrance of protein – protein interactions, and/or increased solution viscosity limiting protein structural movement [20]. However in our study PVP proved to be absolutely unacceptable for lyophilization of ISP phage.

Another stabilizer without any effect appeared to be glycine. Complete phage inactivation immediately after lyophilization was observed with both concentrations of 0.1 and 0.5 M Glycine is important in the lyophilization process mostly as a bulking agent and tonicity adjuster, but at the same time glycine alone has a minimal protective effect because it tends to crystallize during freezing. Glycine as a crystalline bulking agent is more suitable for lyophilization of small-chemical drugs and some peptides [20].

The sugar alcohol mannitol presents also one of the common excipients but mannitol in comparison with other sugars can be easily crystallized and its crystallization is responsible for the destabilization of some proteins during lyophilization. In general mannitol appears most effective in combination with other stabilizers [20]. Freezing rate also influences the extent of crystallization of mannitol which may potentially affect protein stability and reconstitution.

Also in our case, no phage activity was detected after lyophilization in mannitol at a concentration of 0.1 M and a 4 log decrease in phage titer was observed at 0.5 M, caused by the lyophilization procedure itself. This was followed by stable maintenance of the same titer throughout 27 months and a 2 log decrease after a 37 month storage period.

A polyhydric alcohol such as PEG is among the most commonly used and effective cryoprotectants. PEG can be affiliated to two different groups of excipients, i.e., polymers and non-aqueous solvents [21]. Immediately after lyophilisation, a 1.8 and 5.0 log decrease of the ISP titer was detected for 1 and 5% PEG 6000 preparations, respectively. Activity of ISP during storage diminished gradually, resulting in a final 3 log (for 1% PEG 6000) and a 1.7 log pfu/ml (for 5% PEG 6000), after the 37 month storage period.

Nowadays the most popular cryoprotectants are sugars. The mechanism of their cryoprotection implies vitrification during freezing and formation of glass matrix within which phages (in our case) are prevented from aggregation which protects them against mechanical stress of ice crystals [21]. It is generally accepted that among all sugars trehalose is the most preferable cryoprotectant for biomolecules due to its minimal hygroscopicity, the absence of internal hydrogen bounds which allows more flexible formation of hydrogen bonds with proteins during freeze-drying, a very low chemical reactivity and finally, a higher glass transition temperature [21]. In our study, trehalose, along with sucrose, also proved to be the the most effective stabilizer, in particular at a concentration of 0.5 M: approximately only one log decrease was observed as an immediate loss and further on one log decrease was observed at this concentration for both stabilizers after a 37 month storage period.

**Figure 2:**
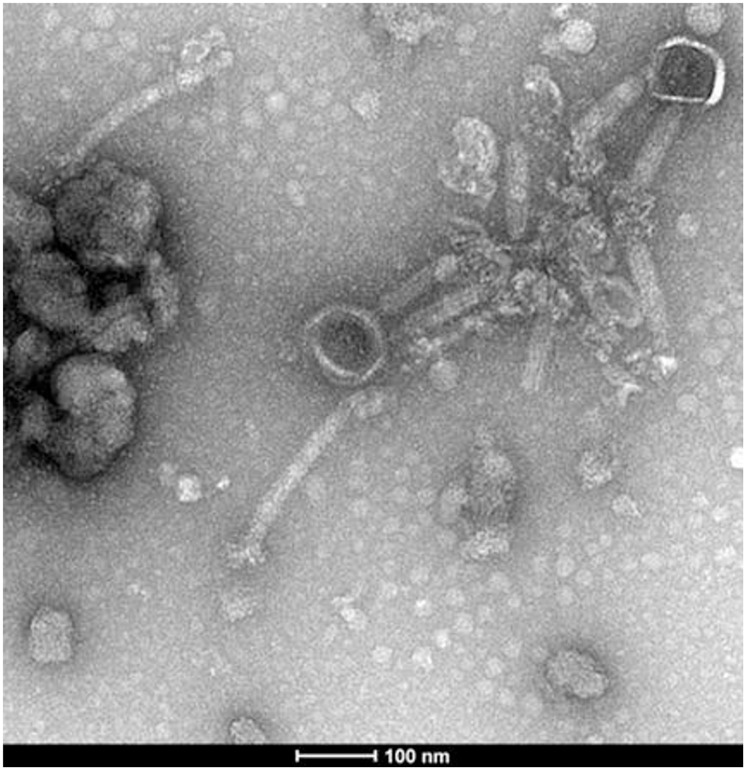
Transmission electron micrograph of lyophilized ISP sample in 0.5 M mannitol.

Transmission electron microscopy of the lyophilized samples showed that in the samples with decreased phage activity number of intact phage particles was drastically reduced due to their complete lysis or the destruction, as depicted by loss of tails and by uranyl-acetate-penetrated heads. The phage particles were associated mostly with bacteriophage debris, forming agglomerates, often by tail-tail interactions. The samples differed from each other mostly by the number of intact phage particles, without other significant specific changes. Therefore Figure 2 presenting ISP lyophilized in mannitol 0.5 M can be considered as an example of the common picture seen in all lyophilized samples.

**Figure 3:**
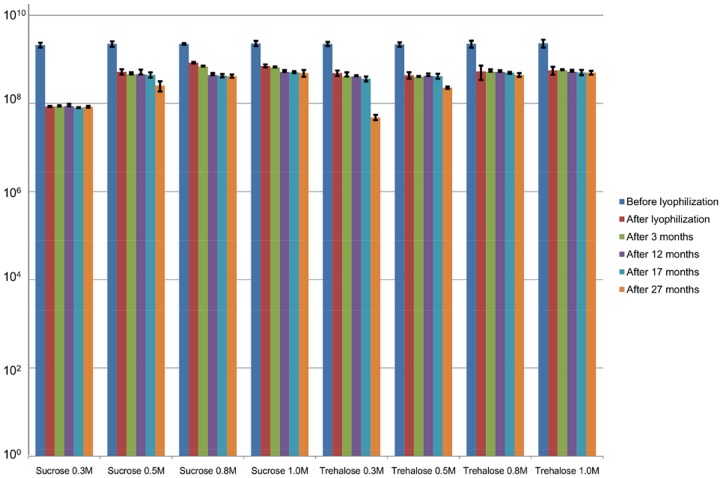
Stability of ISP (9 log pfu/ml) in two different stabilizers after freeze-drying procedure and storage at 4°C. The results are the mean values of three titrations. Standard deviations are indicated.

Based on the results of the first set of experiments, as determined after a 10 month period, the best two out of the six tested stabilizers, i.e. sucrose and trehalose, were chosen for further detailed study. The level of stabilization afforded by sugars generally depends on their concentration [21]. The results of a number of studies [7], [10], [11], [12] show that various concentrations are optimal for different phages. Therefore, four different concentrations of each of these stabilizers, i.e. 0.3, 0.5, 0.8 and 1.0 M were applied (Figure 3). The starting titer of ISP in the second set of experiments was 9 log pfu/ml, instead of 8log pfu/ml as in the first set of experiments. The immediate decrease in titer after lyophilization varied between 0.6 and 1.4 logs and the best results were obtained in case of 0.8 and 1.0 M sucrose with loss of only 0.4–0.5 logs (Figure 3). During the 27 month storage period, the activity of ISP stayed stable with variations within one log in all preparations of sucrose and trehalose, except for 0.3 M of trehalose.

**Figure 4:**
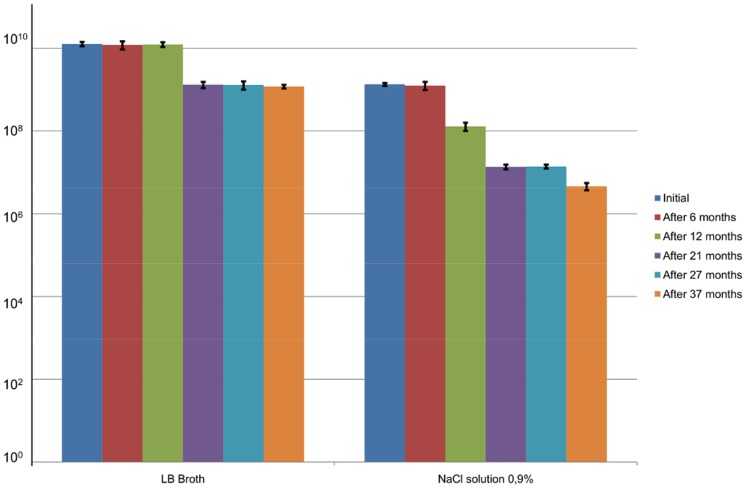
Stability of ISP (10 log pfu/ml) in LB broth and 0.9% NaCl (9 log pfu/ml) at 4°C. The results are the mean values of three titrations. Standard deviations are indicated.

As a control, phage stability was also monitored in LB broth and in physiological saline (0.9% NaCl) for the same period at 4°C (Figure 4). Phages stayed stable in LB broth for one year and a one log decrease was observed only after the 21 month period, while in physiological saline phage activity decreased gradually by each log after 12, 21 and 37 months resulting in a final 6.7 log pfu/ml.

Table 1 summarizes phage lyophilization studies, dating back to at least 1962, and carried out mostly for well-known *E. coli* phages. Duration of storage periods that have been checked ranges from 7 days to 20 years. Comparison of the data is difficult, because very different stabilizers at different concentrations, different initial phage titers and different storage temperatures have been used, and/or because parameters, such as storage temperature, have not been documented and/or titer changes have been expressed in different manners.

Thus far, phages have been lyophilized most frequently in normal culture media with addition of gelatin, peptone and some sugars at different concentrations [10], [11], [12], [13]. It is also important to notice that most of the stabilizers used in the studies at the end of the last century with the aim to optimize phage storage conditions, nowadays are not pharmaceutically acceptable any more. In our study, all stabilizers were chosen taking into account this particular criterion. In a number of studies [10], [11], [12], sugars, in particular sucrose, proved to be effective cryoprotectants for phage lyophilization and one of the best results were obtained in case of 14 phages active against *Corynebacterium* spp., lyophilized in the presence of 10% sucrose [10].

In most of the studies (Table 1), the storage period after lyophilization either is not implied or is limited to several months. However the longest post-lyophilization storage period thus far studied lasted 20 years [22], indicating that the phage particles remained viable, but without specifying the exact titers before and after lyophilization/storage. In three other studies [10], [23], [24] the storage period also comprises several years (from 2 to 18) during which lyophilized phages showed high stability with a maximum 2 log decrease. In two of these studies [23], [24], skim milk was used as a stabilizer and the third study [10] used more complex media, consisting of 20% peptone +10% sucrose +2% sodium glutamate.

Four studies on lyophilization and storage stability involving a relatively large number of phages (from 14 to 25), clearly indicate that phage survival rate also strongly varies from phage to phage and does not necessarily depend on only processing conditions [10], [22], [23], [24]. The phages used in these studies belong to different morphological families and are active against different species of bacteria. Based on all the studies presented in Table 1, it can be assumed that there is no similarity of survival rate even between very closely related phages lyophilized in the same conditions, for e.g. such as T3 and T7 [11]. Therefore, lyophilization conditions must be defined and adjusted for each phage individually which makes pharmaceutical formulations of therapeutically important phages more elaborative especially regarding phage cocktails.

Interesting novel approaches have been presented in several recent studies (Table 1). Anany *et al*. [25] investigated the effectiveness of phages immobilized on cellulose membranes with further application in meat preservation and Alfadhel *et al.* [26] and Puapermpoonsiri *et al*. [6], [7] evaluated the potency of therapeutic phages, encapsulated in biodegradable microspheres, for in vitro application of nasal inserts harboring certain doses of the same phages. All authors implied application of lyophilized phages in their novel formulations and therefore different stabilizers/conditions were tested to define most favorable conditions for maximal phage activity. However, in most of the experiments shelf-life preservation either was not considered at all or was limited to maximum several month period.

### Conclusion

In conclusion, we found that sucrose and trehalose proved to be quite effective stabilizers for lyophilization and long term preservation of bacteriophage ISP. The most efficient concentrations for these stabilizers in this study were 0.8 and 1.0 M with maximal loss of 0.6 log10s after lyophilization procedure and steady stability during a storage period of 27 months. Our findings are also comparable with the results of most studies reviewed here and presented in Table 1 according to which overall titer losses usually range between 1 and 3 logs, *i.e.*, between 90 and 99.9% of the initial titer.

## References

[1] Abedon ST, Kuhl SJ, Blasdel BG, Kutter EM (2011) Phage treatment of human infections. Bacteriophage 1: 66–85. 22334863 10.4161/bact.1.2.15845 PMC3278644

[2] Burrowes B, Harper DR, Anderson J, McConville M, Enright MC (2011) Bacteriophage therapy: Potential uses in the control of antibiotic-resistant pathogens. Exp Rev Anti Infect Ther 9: 775–785. 10.1586/eri.11.90 21905786

[3] Haq IU, Chaudhry WN, Akhtar MN, Andleeb S, Qadri I (2012) Bacteriophages and their implications on future biotechnology: A review. Virol J 9: 9–16. 22234269 10.1186/1743-422X-9-9 PMC3398332

[4] Lu TK, Koeris MS (2011) The next generation of bacteriophage therapy. Curr Opin Microbiol 14: 524–531. 21868281 10.1016/j.mib.2011.07.028

[5] Jikia D, Chkhaidze N, Imedashvili E, Mgaloblishvili I, Tsitlanadze G, et al (2005) The use of a novel biodegradable preparation capable of the sustained release of bacteriophages and ciprofloxacin, in the complex treatment of multidrug-resistant *Staphylococcus aureus*-infected local radiation injuries caused by exposure to Sr90. Clin Exp Dermatol 30: 23–26. 15663496 10.1111/j.1365-2230.2004.01600.x

[6] Puapermpoonsiri U, Ford SJ, van der Walle CF (2010) Stabilization of bacteriophage during freeze drying. Int J Pharm 389: 168–175. 20105458 10.1016/j.ijpharm.2010.01.034

[7] Puapermpoonsiri U, Spencer J, van der Walle CF (2009) A freeze-dried formulation of bacteriophage encapsulated in biodegradable microspheres. Eur J Pharm Biopharm 72: 26–33. 19118627 10.1016/j.ejpb.2008.12.001

[8] Golshahi L, Lynch KH, Dennis JJ, Finlay WH (2011) In vitro lung delivery of bacteriophages KS4-M and ΦKZ using dry powder inhalers for treatment of *Burkholderia cepacia* complex and *Pseudomonas aeruginosa* infections in cystic fibrosis. J Appl Microbiol 110: 106–117. 20875034 10.1111/j.1365-2672.2010.04863.x

[9] Matinkhoo S, Lynch KH, Dennis JJ, Finlay WH, Vehring R (2011) Spray-dried respirable powders containing bacteriophages for the treatment of pulmonary infections. J Pharm Sci 100: 5197–5205. 22020816 10.1002/jps.22715

[10] Carne HR, Greaves RI (1974) Preservation of corynebacteriophages by freeze-drying. J Hyg 72: 467–470. 4602040 10.1017/s0022172400023706 PMC2130534

[11] Cox CS, Harris WJ, Lee J (1974) Viability and electron microscope studies of phages T3 and T7 subjected to freeze-drying, freeze-thawing and aerosolization. J Gen Microbiol 81: 207–215. 4132527 10.1099/00221287-81-1-207

[12] Davies JD, Kelly MJ (1969) The preservation of bacteriophage H1 of *Corynebacterium ulcerans* U103 by freeze-drying. J Hyg 67: 573–583. 5261208 10.1017/s0022172400042030 PMC2130766

[13] Shapira A, Kohn A (1974) The effects of freeze-drying on bacteriophage T4. Cryobiology 11: 452–464. 4615882 10.1016/0011-2240(74)90113-8

[14] Steele PR, Davies JD, Greaves RI (1969) Some factors affecting the viability of freeze-thawed T4 bacteriophage. J Hyg 67: 107–114. 4993555 10.1017/s0022172400041486 PMC2130685

[15] Merabishvili M, Pirnay J-P, Verbeken G, Chanishvili N, Tediashvili M, et al (2009) Quality-controlled small-scale production of a well-defined bacteriophage cocktail for use in human clinical trials. PLoS ONE 4: e4944. 19300511 10.1371/journal.pone.0004944 PMC2654153

[16] Rice P, Longden I, Bleasby A (2000) EMBOSS: The European molecular biology open software suite. Trends Genet 16: 276–277. 10827456 10.1016/s0168-9525(00)02024-2

[17] Vandersteegen K, Mattheus W, Ceyssens PJ, Bilocq F, De Vos D, et al (2011) Microbiological and molecular assessment of bacteriophage ISP for the control of *Staphylococcus aureus* . PLoS ONE 6: e24418. 21931710 10.1371/journal.pone.0024418 PMC3170307

[18] Kwan T, Liu J, DuBow M, Gros P, Pelletier J (2005) The complete genomes and proteomes of 27 *Staphylococcus aureus* bacteriophages. Proc Nat Acad Sci USA 102: 5174–5179. 15788529 10.1073/pnas.0501140102 PMC556006

[19] Kvachadze L, Balarjishvili N, Meskhi T, Tevdoradze E, Skhirtladze N, et al (2011) Evaluation of lytic activity of staphylococcal bacteriophage Sb-1 against freshly isolated clinical pathogens. Microb Biotechnol 4: 643–650. 21481199 10.1111/j.1751-7915.2011.00259.x PMC3819013

[20] Wang W (2000) Lyophilization and development of solid protein pharmaceuticals. Int J Pharm 203: 1–60. 10967427 10.1016/s0378-5173(00)00423-3

[21] Abdelwahed W, Degobert G, Stainmesse S, Fessi H (2006) Freeze-drying of nanoparticles: Formulation, process and storage considerations. Adv Drug Deliv Rev 58: 1688–1713. 17118485 10.1016/j.addr.2006.09.017

[22] Ackermann H-W, Tremblay D, Moineau S (2004) Long-term bacteriophage preservation. World Fed Culture Coll Newslett 38: 35–40.

[23] Clark WA (1962) Comparison of several methods for preserving bacteriophages. Appl Microbiol 10: 466–471. 14021544 10.1128/am.10.5.466-471.1962 PMC1057894

[24] Zierdt CH (1988) Stabilities of lyophilized *Staphylococcus aureus* typing bacteriophages. Appl Environ Microbiol 54: 2590. 2974273 10.1128/aem.54.10.2590-.1988 PMC204327

[25] Anany H, Chen W, Pelton R, Griffiths MW (2011) Biocontrol of *Listeria monocytogenes* and *Escherichia coli* O157:H7 in meat by using phages immobilized on modified cellulose membranes. Appl Environ Microbiol 77: 6379–6387. 21803890 10.1128/AEM.05493-11 PMC3187159

[26] Alfadhel M, Puapermpoonsiri U, Ford SJ, McInnes FJ, van der Walle CF (2011) Lyophilized inserts for nasal administration harboring bacteriophage selective for *Staphylococcus aureus*: In vitro evaluation. Int J Pharm 416: 280–287. 21771648 10.1016/j.ijpharm.2011.07.006

[27] Weiss FA (1957) Maintenance and preservation of cultures. In: Conn HJ, editors. Manual of microbiological methods. New York: McGraw-Hill Book Co Inc. 99–119.

[28] Greaves RIN, Davies JD (1965) Separate effects of freezing, thawing and drying living cells. Ann NY Acad Sci 125: 548–558. 5221080 10.1111/j.1749-6632.1965.tb45413.x

[29] Cox CS, Heckly RJ (1973) Effects of oxygen upon freeze-dried and freeze-thawed bacteria: Viability and free radical studies. Can J Microbiol 19: 189–194. 4572374 10.1139/m73-029

[30] Lion MG, Bergmann ED (1961) The effects of oxygen on freeze-dried *Escherichia coli* . J Gen Microbiol 24: 191–203. 13762526 10.1099/00221287-24-2-191

[31] Zierdt CH (1959) Preservation of staphylococcal bacteriophage by means of lyophilization. Am J Clin Pathol 3: 326–331. 10.1093/ajcp/31.4.326 13637043

